# Osmotic Vesicle Collapse of Sealed Inside–Out Membrane Vesicles From Red Blood Cells

**DOI:** 10.3389/fphys.2021.727726

**Published:** 2021-08-26

**Authors:** Teresa Tiffert, Virgilio L. Lew

**Affiliations:** Physiological Laboratory, Department of Physiology, Development and Neuroscience, University of Cambridge, Cambridge, United Kingdom

**Keywords:** red blood cells, inside–out membrane vesicles, electron microscopy of membrane vesicles, membrane transport, vesicle collapse

## Abstract

The preparation of plasma membrane vesicles from a large variety of cells has contributed a wealth of information on the identity and vectorial properties of membrane transporters and enzymes. Vesicles from red blood cell (RBC) membranes are generated in media of extremely low tonicity. For functional studies, it is required to suspend the vesicles in higher tonicity media in order to bring the concentrations of the substrates of transporters and enzymes under investigation within the physiological ranges. We investigated the effects of hypertonic transitions on the vesicle morphology using transmission electron microscopy. The results show that hypertonic transitions cause an irreversible osmotic collapse of sealed membrane vesicles. Awareness of the collapsed condition of vesicles during functional studies is critical for the proper interpretation of experimental results.

## Introduction

The original method for generating inside–out vesicles (IOVs) from red blood cell (RBC) plasma membranes was developed by Steck et al. (1970) and Steck and Kant (1974). The initial step was to lyse the RBCs in large volumes of low-osmolarity, low-ionic strength media free of divalent cations, lightly buffered to pH 7.5–7.8. Further lengthy washes and incubations of the cell membranes in this medium at low temperature were shown to cause disassembly of their underlying cortical cytoskeleton, leaving naked membranes easily vesiculated by vigorous shearing forces.

In the IOVs generated by the Steck–Kant method, the activity of the Ca^2+^-activated K^+^ channel (Gardos channel) was reported absent or inactivated (Sarkadi et al., 1980). Searching for the stage at which channel activity might have been lost, we discovered that incubating membranes from recently lysed cells at 37°C in the Steck–Kant lysis medium elicited rapid and *spontaneous* inside–out vesiculation during cytoskeletal disassembly (Lew et al., 1982; Lew et al., 1988; Tiffert and Lew, 2014). We labeled the vesicles generated by this rapid method as “one-step IOVs.” This discovery enabled us to follow the membrane changes during spontaneous vesiculation in organized samples, leading to the elucidation of the dynamic morphology of the process and the mechanism of vesiculation (Lew et al., 1988; Tiffert and Lew, 2014).

**FIGURE 1:**
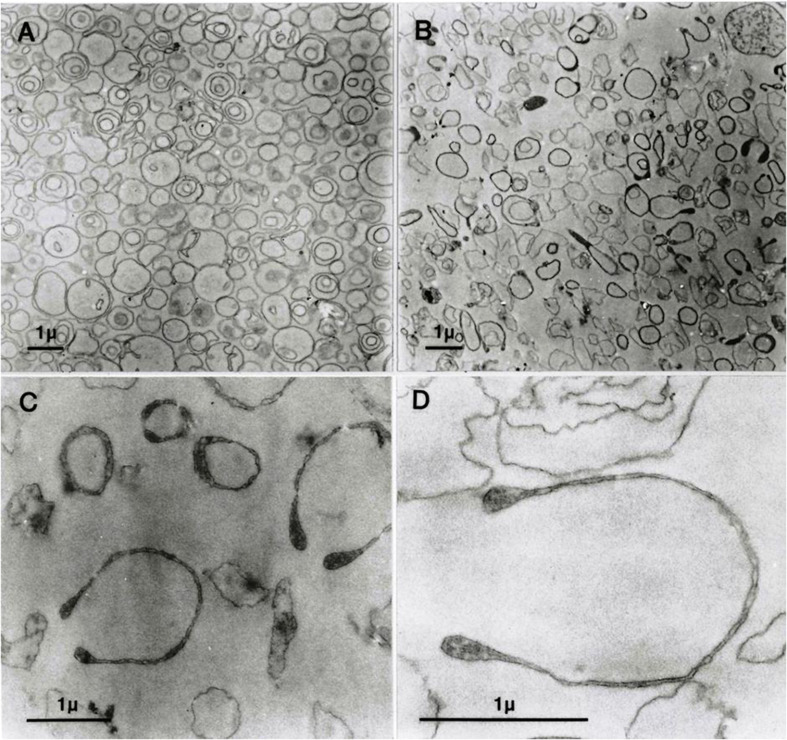
Transmission electron micrographs (EMs) of predominantly inside–out vesicles obtained by the one-step method (Lew et al., 1982; Garcia-Sancho and Alvarez, 1989). **(A)** Aliquots of the vesicle suspension after incubation in lysis medium A at 37°C were treated and further processed for EM as outlined in Section “Materials and Methods”. **(B–D)** Vesicles in aliquots of the suspension described for panel A were spun, washed, and resuspended in medium B, processed for EM, and observed at increasing magnifications in **(C,D)**.

Vesicles generated by the “one-step” method show a remarkable heterogeneity of single, double, or triple concentric vesicles (Lew et al., 1988), as also shown in the electron micrograph of Figure 1A. Functional studies documented that the one-step vesicles retained the activity of the Na/K pump, plasma membrane calcium pump, Gardos channel, and anion exchanger, i.e., the main ion transporter of the RBC membrane (Garcia-Sancho et al., 1982; Lew et al., 1982; Alvarez and García-Sancho, 1987; Garcia-Sancho and Alvarez, 1989).

However, the effects of the hypertonic transitions required for functional studies on the morphology of the one-step IOVs have never been reported earlier. This is the subject of the current investigation.

## Materials and Methods

The protocol for the preparation of the one-step vesicles used in this study was comprehensively reported earlier (Garcia-Sancho et al., 1982; Lew et al., 1982; Garcia-Sancho and Alvarez, 1989) and was succinctly outlined in the study by Tiffert and Lew (2014; Figure 1). The composition of the hypotonic medium in which the RBCs were lysed and the vesicles were formed (medium A) was (in mM): HEPES-Na (pH 7.5) 2.5 and EGTA 0.1. The composition of the medium used for transport studies in the past (medium B) (Lew et al., 1982; Garcia-Sancho and Alvarez, 1989), which was applied here for resuspending the freshly prepared vesicles to investigate the morphological effects of the hypertonic transition, was (in mM): NaCl, 50; KCl, 10; Na-HEPES (pH 7.5), 25; MgCl_2_, 1.5; EGTA, 0.02.

### Preparation of Samples for Electron Microscopy (EM)

Immediately after the incubation in lysis medium A at 37°C, aliquots of the vesicle suspension were transferred to 1.5-mL microfuge tubes and spun at 10,000 × *g* for 1 min. The loosely pelleted vesicles were postfixed in 1% OsO_4_ and processed as reported earlier (Lew et al., 1985; Lew et al., 1988). For testing the hypertonicity effects, the vesicles in some of the aliquots were resuspended in medium B and incubated for 10–30 min at 37°C. After incubation, the vesicles were spun at 10,000 × *g* for 1 min in their original microfuge tubes, and the pellets were postfixed in 1% OsO_4_ and processed similar to the samples in medium A.

## Results

Figure 1A shows freshly formed vesicles in hypotonic medium A with the mixture of single, double, and triple concentric vesicles typical of this preparation (Lew et al., 1988).

Figure 1B shows the change in appearance generated by suspending the freshly formed vesicles in medium B. Vesicle membranes are clearly divided into two distinct populations with thin or thick membrane outlines. Some of the thick membrane vesicles configure headphone shapes.

When vesicles suspended in medium B (Figure 1B) are washed and resuspended back in medium A, their appearance reverts as shown in Figure 1A. This was the only procedure found to be capable of reversing the effects of hypertonic exposure.

**FIGURE 2:**
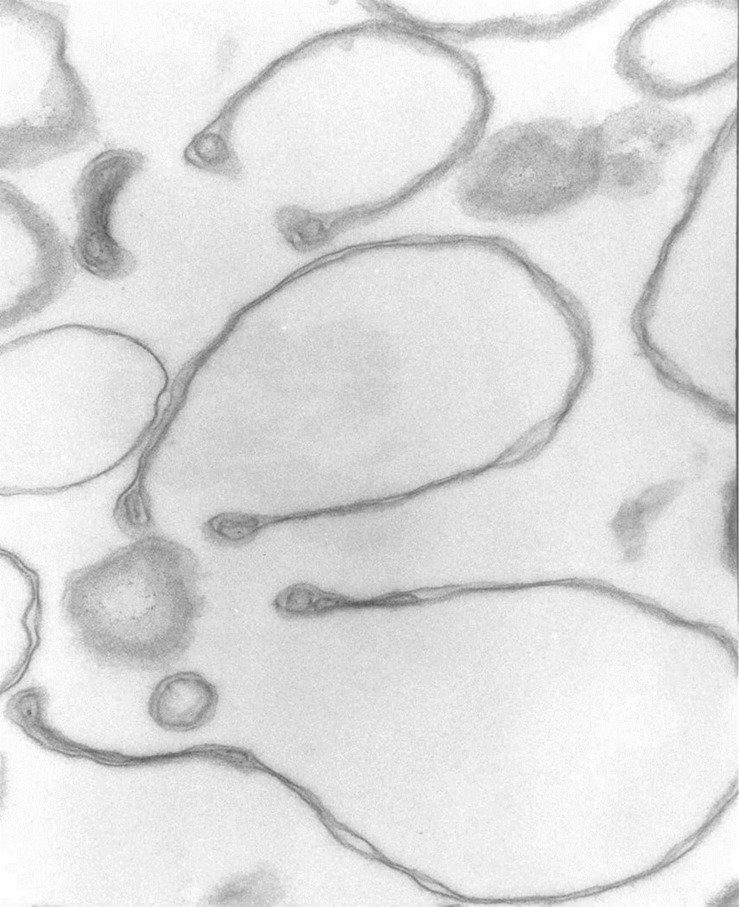
Headphone shape samples. Transmission electron micrograph of selected collapsed vesicles showing the close apposition of the cytoskeleton-free membranes outlining the “headphone” shapes in longitudinal sections. Note the massive reduction in the intra-vesicular space, the outline of the 3D cup shape of the collapsed vesicles, and the displacement of contents toward the rim of the cup. 67,500x.

**FIGURE 3:**
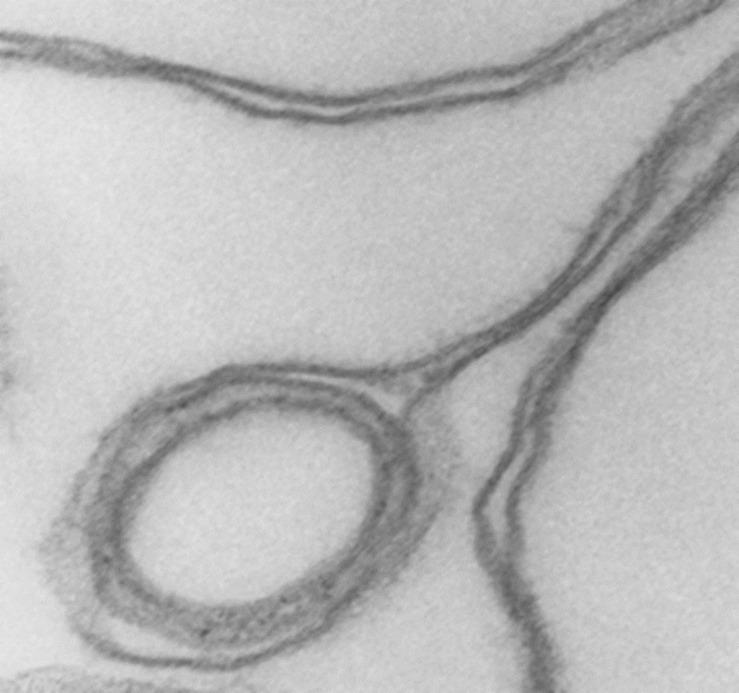
Detail of cup rim and double membranes. Transmission electron micrograph showing details of membrane apposition proximities in collapsed vesicles and of a small internal vesicle with a collapsed double membrane displaced toward the rim of the larger host vesicle shaping the bulge of the rim at that point. 175,000x.

At higher magnifications, the thick membrane outlines in Figure 1B can be seen to result from double membranes in close apposition (Figures 1C,D, 2, 3). Figure 1D offers a direct comparison between thin and thick membrane outlines. The headphone configuration combined with the close double-membrane apposition evokes the shape of a fully deflated ball or double cup as seen in a longitudinal section. This exposes the three-dimensional (3D) cup-like shape of the double-membrane vesicles behind their headphone appearance in longitudinal sections. Transverse sections would be expected to render thick circular images as shown in Figure 1C. Figures 2, 3 show how internalized membranes from original double and triple concentric vesicles become displaced toward the thick rim of the cups.

## Discussion

The results in Figure 1 show that in heterogeneous populations of plasma membrane vesicles from human RBCs, a substantial fraction of the vesicles experiences a drastic volume collapse when transferred to media of increased osmolarity (Figures 1B–D, 2). Only sealed vesicles with retained water permeability and restricted solute permeability can experience such a collapse under osmotic gradients. Hence, the heterogeneous morphological response to hypertonic transitions segregates vesicles by their sealed condition.

Cytoskeleton-free RBC membranes were shown to behave like a two-dimensional liquid devoid of intrinsic shape control (Tiffert and Lew, 2014). The curvature along the rim of the cup-shaped collapsed vesicles, a sample of which is shown in more detail in Figure 3, is determined by a combination of the intrinsic bending modulus of the free membrane (Evans, 1983), membrane protein interactions on the membrane curvature (Reynwar et al., 2007), and the bulge created by internal vesicles displaced toward the rim during the volume collapse of the host vesicle. In retrospect, casual microscopic sightings of “sharper bordered” vesicles in medium B were noted during past functional studies (Lew et al., 1982), but not interpreted at time. This opens the possibility of estimating the proportion of collapsed vesicles from microscopic observations or light-scattering measurements.

Figures 1C,D, 2 reveal that the condition of sealed vesicle subpopulations during transport experiments is that of a greatly reduced intravesicular volume and a massive increase in area/volume ratio relative to their original spherical condition. Rate constants used for estimates of membrane permeabilities in vesicles are heavily influenced by their area/volume ratio. It is therefore essential to be aware of the osmotic collapsed condition of the sealed vesicles exposed to the usual hyperosmotic transitions applied in functional studies for the correct interpretation of kinetic data (Lew et al., 1983; Alvarez et al., 1988).

## Data Availability Statement

The original contributions presented in the study are included in the article/supplementary material, further inquiries can be directed to the corresponding author/s.

## Author Contributions

Both authors contributed equally to all aspects of this research.

## Conflict of Interest

The authors declare that the research was conducted in the absence of any commercial or financial relationships that could be construed as a potential conflict of interest.

## Publisher’s Note

All claims expressed in this article are solely those of the authors and do not necessarily represent those of their affiliated organizations, or those of the publisher, the editors and the reviewers. Any product that may be evaluated in this article, or claim that may be made by its manufacturer, is not guaranteed or endorsed by the publisher.
